# Psychometric performance of the WHOQOL-HIV BREF among people living with HIV on antiretroviral therapy in Bauchi State, Nigeria

**DOI:** 10.1371/journal.pone.0338095

**Published:** 2025-12-12

**Authors:** Ekerette Emmanuel Udoh, Adesola Zaidat Musa, Tubosun Alex Olowolafe, Taiwao Mofadeke Jaiyeola

**Affiliations:** 1 Department of Public Health, Faculty of Basic Medical and Health Sciences, Lead City University, Ibadan, Nigeria; 2 Society for Family Health, Abuja, Nigeria; 3 Nigerian Institute of Medical Research, Lagos, Nigeria; 4 Department of Epidemiology and Medical Statistics, College of Medicine, University of Ibadan, Ibadan, Nigeria; Hue University of Medicine and Pharmacy, VIET NAM

## Abstract

**Background:**

The well-being of people living with HIV (PLHIV) remains a significant public health concern. Despite advancements in ART, PLHIV face various challenges impacting their quality of life (QOL). Measuring the QOL of PLHIV is essential for improving care, shaping policy, and evaluating intervention. However, the extent to which the measurement properties of the World Health Organization Quality of Life HIV BREF (WHOQOL-HIV BREF) tool, in the Nigeria context is limited. This study evaluated the psychometric performance of the WHOQOL-HIV BREF in a Nigerian population, with the aim of examining its construct validity, reliability, and sensitivity when used to assess the well-being of PLHIV.

**Methods:**

This was a cross-sectional quantitative study involving of 790 PLHIV aged 18 years and older in Bauchi State, Nigeria. Data was collected between May and October 2023. Quality of life was assessed using the 31-item WHOQOL-HIV BREF which assesses QOL based on the 6 domains, including Physical, Psychological, Level of Independence, Social Relationships, Environmental, and Spirituality/Religion/Personal Beliefs. The general quality of life and general health perception was also assessed. The psychometric evaluation of the instrument in this population included analyses of internal consistency reliability, confirmatory factor analysis (CFA) to assess construct validity, and sensitivity analysis to examine measurement responsiveness.

**Results:**

Confirmatory factor analysis of the WHOQOL-HIV BREF in the sample indicated poor model fit (CFI = 0.807, TLI = 0.767, RMSEA = 0.110, SRMR = 0.136), indicating that the original six-domain structure was not well supported in the study population and context. Internal consistency (Cronbach’s alpha) varied widely across domains, ranging from very low to acceptable: Physical Health (α = 0.386), Psychological Health (α = 0.722), Level of Independence (α = 0.531), Social Relationships (α = 0.693), Environment (α = 0.775), and Spirituality (α = 0.304). The Global Quality of Life score demonstrated high reliability (α = 0.884). Nonetheless, high scores were observed for general quality of life (Mean = 3.86) and health perception (Mean = 3.65), as well as for independence (Mean = 14.84) and social relationships (Mean = 14.93). Moderate scores were found in physical health (Mean = 13.99), psychological health (Mean = 13.95), environmental (Mean = 12.74), and spiritual well-being (Mean = 13.77).

**Conclusions:**

This study revealed limited structural validity and varied internal consistency in this sample, with some domains of the WHOQOL-HIV BREF performing poorly. These findings reinforce concerns raised by researchers about the use of the tool without proper contextual adaptation. Further research is needed to better understand QOL in the Nigeria population, by refining and validating the QOL assessment tools for the country, to ensure adequate monitoring of the health of PLHIV.

## Introduction

Quality of life is a complex and multi-dimensional concept that is influenced by a range of physical, psychological, and social factors [1,2]. People living with HIV (PLHIV) often face challenges related to their physical health, social stigma, and financial hardship, which can negatively impact their quality of life [3–5]. Studies have shown that people living with HIV often experience decreased quality of life, particularly in the areas of physical and mental health, compared to the general population [6,7]. This is due partly to the long-term nature of HIV, the toxicities associated with antiretroviral therapy, and the presence of other medical conditions, such as cardiovascular diseases, liver disease, and certain cancers, that are more common in PLHIV [6,8].

The term Quality of life (QOL), as defined by the World Health Organization (WHO), refers to individuals’ perception of their position in life in the context of the culture and value systems in which they live, and in relation to their goals, expectations, standards, and concerns [9]. This broad definition underscores that QOL is subjective, influenced not only by physical health but also by how individuals perceive their life within a society. While ART has significantly improved the quality of life for many PLHIV by reducing viral load and preventing opportunistic infections [10], challenges persist. PLHIV continue to experience difficulties related to chronic illness management [11], stigma, and social discrimination [12], which can exacerbate feelings of isolation and mental health struggles. The long-term impacts of HIV and its treatment require continuous research to better understand and address these issues.

The WHOQOL-HIV BREF (Brief version of the WHO Quality of Life HIV instrument) is a validated tool designed to assess the quality of life specifically among people living with HIV [13]. The instrument evaluates the multiple dimensions of QOL, including physical health, psychological well-being, social relationships, spiritual, and environment. It provides insights into the challenges PLHIV face in these areas and helps to identify possible interventions for improving their health and quality of life. The WHOQOL-HIV BREF is a relevant tool as it provides a reliable and comprehensive way to measure QOL in the context of HIV. This tool has been widely used in various global settings to assess the impacts of HIV and its treatment, making it an invaluable resource for understanding the experiences of PLHIV [14]. However, literature reveals that deviations or misalignments from the six domains may occur due to cultural and contextual factors. For instance, a previous study demonstrated and emphasized that cultural values significantly influence how individuals perceive and respond to questions, potentially affecting the validity of domains such as spirituality and social relationships [15].

In Nigeria, although several studies have used the WHOQOL-HIV BREF to report QOL levels among PLHIV, they typically do not explore the underlying measurement performance [16–18]. Many of these studies lack the rigorous psychometric evaluations, such as construct validity testing or assessments of floor/ceiling effects, that are crucial for determining whether the instrument functions as intended in the Nigerian context. By contrast, a recent study examined the factor structure of the adapted WHOQOL-BREF among Nigerian adolescents without HIV and found a misalignment with its original four-domain structure, with a two-factor model providing a better fit. This suggests that cultural and developmental factors may influence how QOL is perceived and measured. Although the study did not focus on PLHIV, it buttresses the potential for deviation or misalignment in the structure and interpretation of QOL instruments, highlighting the need for careful validation within specific populations and contexts. A different large-scale study in France also reported deviations in the expected domain structure of the WHOQOL-HIV BREF, highlighting the need for context-specific validation even in high-resource settings [19]. In line with this, the WHO advises against merely translating its QOL instruments into new languages without first ensuring cultural adaptation. Researchers have also stressed that adapting QOL tools across cultures is essential, as the concept of quality of life can vary significantly across different sociocultural settings [20,21].

The Nigerian National Strategic Framework on HIV (2021–2025) had an ambitious goal of fast-tracking the national response to end AIDS as a public health threat by 2030 [22]. While progress has been made in expanding access to ART and reducing new infections, persistent barriers remain. Achieving this goal requires not only biomedical solutions, but also a deeper understanding of the lived experiences and quality of life of PLHIV. However, the assessment of QOL must be carried out with the consideration of the sociodemographic, language, and cultural context of the population. This implies that current and available assessment tools, may be limited in their capabilities in adequately measuring the QOL of individuals in the context of Nigeria. This aligns with the objectives of the current study, which seeks to investigate the psychometric performance of the of the WHOQOL-HIV BREF in the Nigeria context. By utilizing the WHOQOL-HIV BREF, this study aims to provide relevant evidence that can help guide validation and contextual adaptation of the instrument. Ultimately, the findings will contribute to improving the accuracy and cultural relevance of QOL assessments for PLHIV in Nigeria.

## Methods

### Study settings

This study was a cross-sectional quantitative study involving a survey of a sample of 790 male and female PLHIV aged 18 years and older, as well as data extraction from clinical records of PLHIV currently receiving ART at two public treatment facilities in two Local Government Areas (LGAs) in Bauchi State. Bauchi state is in the North-East geopolitical zone of Nigeria, and has a forecasted population estimate of 6,537,314 as of 2016 [23]. The state has an HIV prevalence of 0.4% compared to the national prevalence of 1.5% [24]. Potential participants for the study were recruited by the healthcare provider in the study facility and were referred to trained data collectors who conducted informed consent process and interviews using a pretested structured questionnaire. Participants were recruited consecutively as they visited the facility for their hospital appointment until the desired sample size was achieved. Data collection spanned 6 months from May to October 2023.

### Measures

Quality of Life, was assessed using the WHO Quality of Life HIV BREF (WHOQOL-HIV BREF) [25], a 31-item instrument designed to measure the quality of life in individuals with HIV infection. The WHOQOL-HIV BREF is derived from the WHO-100 instrument and focuses specifically on the quality of life of people living with HIV/AIDS [25]. The tool encompasses questions covering various domains, including Physical, Psychological, Level of Independence, Social Relationships, Environmental, and Spirituality/Religion/Personal Beliefs. Each domain comprises multiple items, and respondents rate their perception of different aspects of their life within each domain on a Likert scale. The Likert scale ranges from 1 to 5, with higher scores indicating better quality of life. This instrument provides a comprehensive assessment of the different facets of quality of life experienced by individuals living with HIV/AIDS. In the WHOQOL-HIV BREF the scoring involved calculating domain scores by summing the item scores within each domain [25]. There is no total score for the instrument. These domain scores provide an overall assessment of the individual’s quality of life across different dimensions. The total expected score for each domain as applied in this study are as follows; Physical Health – 4 items (range: 4–20), Psychological Health – 5 items (range: 5–25), Level of Independence – 4 items (range: 4–20), Social Relationships – 4 items (range: 4–20), Environment – 8 items (range: 8–40), and Spirituality/Religion/Personal Beliefs – 4 items (range: 4–20). A higher score corresponds to better quality of life.

In the survey additional information on sociodemographic variables, external risk factors such as smoking, alcohol and drug abuse, risky sexual behaviours, physical activity, and HIV related factors, were elicited. Information on Internal risk factors (Family history of disease) of the PLHIV was also elicited.

### Data management and analysis

Data management, analysis, and visualization was done using R: A Language and Environment for Statistical Computing (version 3.6.1) [26]. The sensitivity of the WHOQOL-HIV BREF instrument was examined by evaluating the floor and ceiling effects. The floor and ceiling effects were examined to assess the extent to which the responses clustered at the lowest and highest possible scores, respectively. In this study, the floor or ceiling effect were computed as 95% Confidence Interval for the data. The floor of the mean was computed as: , where the floor value represents the lower bound of the 95% confidence interval for the data, indicating that a significant portion of respondents scored at the upper extreme of the scale. The ceiling of the mean was computed as: , where the ceiling value represents the upper bound of the 95% confidence interval for the data, indicating that a significant portion of respondents scored at the lower extreme of the scale. The Cronbach’s Alpha coefficient for the different domains of the tool were assessed to determine the internal construct validity of the QOL tool. The acceptable range for Cronbach’s Alpha is from 0.70 to 0.95 [27].

Descriptive statistics such as frequencies, percentage, mean, median with the spread of the estimates presented using standard deviation, as applicable to summarize all variables. Confirmatory factor analysis (CFA) executed through structural equation modelling (SEM) was employed to test the hypothesis of the original 6 domains of the WHOQOL-HIV BREF, examining their structure and measurement model’s fit to the data collected from PLHIV respondents. In the analysis, an iterative process was undertaken to refine the model by examining the modification indices of the previously constructed models. During the initial model evaluation, any observed (indicator/ manifest) variables that had factor loadings of less than 0.2 with their respective latent variables were removed from the model. It has been recommended that items with low factor loadings (e.g., below 0.2 or 0.3) be removed from the instrument [28]. The model fit of the data were examined using the following fit indices: Comparative Fit Index (CFI), Tucker-Lewis Index (TLI), Root Mean Square Error of Approximation (RMSEA), and Standardized Root Mean Square Residual (SRMR). The following thresholds is typically recommended according to Hu and Bentler as indicative of a good fit: CFI > 0.95, TLI > 0.95, RMSEA < 0.06, and SRMR < 0.08. In addition, the error covariances between observed variables were examined by evaluating their modification indices.

### Ethical concerns

Ethical approval was sought from the Bauchi State Health Research Ethics Committee (BASHREC), with Approval No: NREC/03/11/19B/2021/018) for the implementation of the study in the state. The study only included participants who willingly provided written informed consent to be surveyed. Permission to conduct the study was obtained from the proprietors of the health facilities in the two study LGAs in the State.

## Results

Table 1 and Table 2 presents the demographic characteristics and the health behavior and medical history of the participants. Of the total 790 participants in the survey about 73% were females, and overall age of all participants was 34 years (IQR: 29, 40) with majority of participants attaining a secondary education (37%). Majority of the participants were married, with significant proportion being males. The median household income for females was 40,000 Naira (IQR: 25,000–50,000), approximately 25 USD, while for males it was slightly higher at 45,000 Naira (IQR: 35,000–60,000), or about 28 USD.

**Table 1:** Demographic characteristics of survey population.

Characteristic	Overall, N = 790^1^	Female, N = 576^1^	Male, N = 214^1^	p-value^2^
**Age in years**	34 (29, 40)	33 (28, 38)	36 (30, 43)	<0.001
**Educational status**				0.001
* No formal education*	209 (26%)	172 (30%)	37 (17%)	
* Primary education*	203 (26%)	140 (24%)	63 (29%)	
* Secondary education*	289 (37%)	208 (36%)	81 (38%)	
* Tertiary education*	89 (11%)	56 (9.7%)	33 (15%)	
**Occupation**				<0.001
* Professional*	35 (6.6%)	16 (4.6%)	19 (10%)	
* Skilled*	245 (46%)	123 (36%)	122 (66%)	
* Unskilled*	252 (47%)	207 (60%)	45 (24%)	
**Marital status**				<0.001
* Married*	325 (41%)	191 (33%)	134 (63%)	
* Separated or Widowed*	172 (22%)	162 (28%)	10 (4.7%)	
* Single*	293 (37%)	223 (39%)	70 (33%)	
**Have children**	503 (64%)	363 (63%)	140 (65%)	0.5
**Number of children**	3.00 (2.00, 5.00)	3.00 (2.00, 4.00)	5.00 (3.00, 7.00)	<0.001
**Number of children (grouped)**				<0.001
* 1–2 children*	173 (34%)	141 (39%)	32 (23%)	
* 3–4 children*	159 (32%)	132 (36%)	27 (19%)	
* 5 and more*	171 (34%)	90 (25%)	81 (58%)	
**Living together with children**	328 (65%)	202 (56%)	126 (90%)	<0.001
**Approximate total household income**	40,000 (30,000, 55,000)	40,000 (25,000, 50,000)	45,000 (35,000, 60,000)	<0.001
**Approximate total household income (grouped)**				<0.001
* 30,000 Naira and below*	266 (34%)	214 (37%)	52 (24%)	
* Above 30,000 Naira*	524 (66%)	362 (63%)	162 (76%)	

^1^Median (IQR); n (%).

^2^Wilcoxon rank sum test; Pearson’s Chi-squared test.

**Table 2:** Health behaviour and medical history of the survey population.

Characteristic	Overall, N = 790^1^	Female, N = 576^1^	Male, N = 214^1^	p-value^2^
**Ever hospitalized because of HIV disease**	48 (6.1%)	37 (6.4%)	11 (5.1%)	0.5
**Disclosed HIV status**	590 (75%)	424 (74%)	166 (78%)	0.3
**Ever had sex**	783 (99%)	573 (99%)	210 (98%)	0.091
**Number of sexual partners in past 6 months**				<0.001
* More than one*	552 (70%)	380 (66%)	172 (80%)	
* One*	238 (30%)	196 (34%)	42 (20%)	
**Condom used during last sexual intercourse**	561 (71%)	418 (73%)	143 (67%)	0.11
**Frequency of condom use**				0.081
* Always*	408 (52%)	308 (53%)	100 (47%)	
* Not at all*	74 (9.4%)	57 (9.9%)	17 (7.9%)	
* Sometimes*	308 (39%)	211 (37%)	97 (45%)	
**Ever had or been diagnosed of STI before HIV diagnosis**	479 (61%)	347 (60%)	132 (62%)	0.7
**Smoking cigarette**				<0.001
* Formerly smoke*	155 (21%)	111 (21%)	44 (21%)	
* Never smoke*	436 (58%)	339 (63%)	97 (46%)	
* Regular occasional smoker*	159 (21%)	91 (17%)	68 (33%)	
**Frequency of drinking alcohol**				0.035
* Daily or Nearly Daily >4 times/week*	126 (16%)	87 (15%)	39 (18%)	
* Never*	565 (72%)	423 (73%)	142 (66%)	
* Some/ Month 1–3 times/ month*	40 (5.1%)	22 (3.8%)	18 (8.4%)	
* Some/Week 1–4 times/ week*	59 (7.5%)	44 (7.6%)	15 (7.0%)	
**Drank alcohol in the last 12 months**	210 (96%)	147 (99%)	63 (90%)	0.005
**Ever used illicit drugs**				0.019
* Never*	629 (84%)	465 (86%)	164 (80%)	
* Yes and I currently use it now*	55 (7.4%)	31 (5.7%)	24 (12%)	
* Yes but I used it in the past*	61 (8.2%)	45 (8.3%)	16 (7.8%)	
**Illicit drug use in the last month**				0.003
* Daily or Nearly Daily >4 times/w eek*	41 (35%)	18 (24%)	23 (58%)	
* Never*	35 (30%)	25 (33%)	10 (25%)	
* Sometime/ Month 1–3 times/ month*	8 (6.9%)	7 (9.2%)	1 (2.5%)	
* Sometime/Week 1–4 times/ week*	32 (28%)	26 (34%)	6 (15%)	
**Clinic where ART is received far from current residence**	621 (79%)	458 (80%)	163 (76%)	0.3
**Ever experienced stigma because of HIV status**	229 (29%)	185 (32%)	44 (21%)	0.001
**Frequency of exercising**				<0.001
* Daily or Nearly Daily >4 times/w eek*	23 (2.9%)	13 (2.3%)	10 (4.7%)	
* Never*	608 (77%)	466 (81%)	142 (66%)	
* Sometime/ Month 1–3 times/ month*	95 (12%)	65 (11%)	30 (14%)	
* Sometime/Week 1–4 times/ week*	64 (8.1%)	32 (5.6%)	32 (15%)	
**History of Asthma in family**	127 (16%)	92 (16%)	35 (16%)	0.9
**History of Hypertension in family**	206 (26%)	151 (26%)	55 (26%)	0.9
**History of diabetes in family**	152 (19%)	101 (18%)	51 (24%)	0.046
**History of cancer in family**	58 (7.3%)	39 (6.8%)	19 (8.9%)	0.3
**History of depression in family**	68 (8.6%)	52 (9.0%)	16 (7.5%)	0.5

^1^n (%)

^2^Pearson’s Chi-squared test; Fisher’s exact test

Almost all participants (99%) reported ever having sex, with a significantly higher proportion of males reporting having more than one sexual partner in the past 6 months compared to females (66% females vs 80% males; p < 0.001). Additionally, males were less likely to use condoms during their last sexual intercourse compared to females, although this difference was not statistically significant. About 61% of the total sample reported having had or been diagnosed with a STI before their HIV diagnosis – among these, 60% were female and 62% were male. Significant differences were observed in smoking cigarette habits between genders (p < 0.001). High proportion of females reported experiencing HIV-related stigma, while males reported higher levels of exercise frequency.

### Descriptive results of quality of life

The result in Table 3 provides descriptive statistics for each domain of quality of life measured by the WHO-QOL BREF. The domains include General Quality of Life, General Health Perception, Physical Health, Psychological Health, Level of Independence, Social Relationships, Environment, and Spirituality. The mean scores for all items ranged from 2.44 to 4.06, indicating moderate to high levels of quality of life across the different aspects measured. The standard deviations varied from 0.66 to 3.71, suggesting some degree of variability in responses within each domain.

**Table 3:** Descriptive statistics and reliability of WHO-QOL BREF of PLHIV.

Quality of Life Domains	Mean	SD	95% CI Lower Bound (Floor)	95% CI Upper Bound (Ceiling)	Floor Effect	Ceiling Effect	Floor/Ceiling Effect	Reliability (Cronbach’s Alpha)
								
*General QOL*	3.86	0.69	2.51	5.21	No	Yes	No	
*General health perception*	3.65	0.87	1.94	5.36	No	Yes	No	
**Physical Health**	**13.99**	**3.29**	**7.54**	**20.44**	**No**	**Yes**	**No**	**0.386**
* Q10. Pain and discomfort*	3.48	1.65	0.25	6.71	Yes	Yes	Yes	
* Q11. Energy and fatigue*	3.37	1.61	0.21	6.53	Yes	Yes	Yes	
* Q21. Sleep and rest*	3.45	0.99	1.51	5.39	No	Yes	No	
* Q28. Symptoms of PLWHAs*	3.69	0.87	1.98	5.40	No	Yes	No	
**Psychological Health**	**13.95**	**2.84**	**8.38**	**19.52**	**No**	**Yes**	**No**	**0.722**
* Q13. Positive feelings*	3.39	1.48	0.49	6.29	Yes	Yes	Yes	
* Q18. Cognitions*	3.09	0.77	1.58	4.60	No	No	No	
* Q22. Body image and appearance*	3.76	0.94	1.92	5.60	No	Yes	No	
* *Q31. Self-esteem	4.05	0.87	2.34	5.76	No	Yes	No	
* *Q38. Negative feelings	3.14	1.09	1.00	5.28	No	Yes	No	
**Level of Independence**	**14.84**	**2.35**	**10.23**	**19.45**	**No**	**Yes**	**No**	**0.531**
* Q12. Dependence on medication or treatment*	3.08	1.71	−0.27	6.43	Yes	Yes	Yes	
* Q27. Mobility*	3.97	0.66	2.68	5.26	No	Yes	No	
* Q29. Activities of daily living*	3.83	0.82	2.22	5.44	No	Yes	No	
* Q30. Work capacity*	3.95	0.75	2.48	5.42	No	Yes	No	
**Social Relationships**	**14.93**	**2.52**	**9.99**	**19.87**	**No**	**Yes**	**No**	**0.693**
*Q24. Social inclusion*	3.54	0.85	1.87	5.21	No	Yes	No	
*Q32. Personal relationships*	3.84	0.85	2.17	5.51	No	Yes	No	
*Q33. Sexual activity*	3.93	0.98	2.01	5.85	No	Yes	No	
*Q34. Social support*	3.62	0.78	2.09	5.15	No	Yes	No	
**Environment**	**12.74**	**2.61**	**7.62**	**17.86**	**No**	**Yes**	**No**	**0.775**
* Q19. Physical safety and security*	3.47	0.79	1.92	5.02	No	Yes	No	
* Q20. Physical environments*	3.37	0.79	1.82	4.92	No	No	No	
* Q23. Financial resources*	2.44	1.16	0.17	4.71	Yes	No	No	
* Q25. New information or skills*	3.38	0.80	1.81	4.95	No	No	No	
* Q26. Recreation and leisure*	2.99	1.06	0.91	5.07	Yes	Yes	Yes	
* Q35. Home environment*	3.71	0.91	1.93	5.49	No	Yes	No	
* Q36. Health and social care*	3.47	1.30	0.92	6.02	Yes	Yes	Yes	
* Q37. Transport*	2.66	1.38	−0.04	5.36	Yes	Yes	Yes	
**Spirituality**	**13.77**	**3.71**	**6.50**	**21.04**	**No**	**Yes**	**No**	**0.304**
* Q14. Spirituality, Religion, Personal belief*	3.68	1.59	0.56	6.80	Yes	Yes	Yes	
* Q15. Forgiveness*	4.06	1.35	1.41	6.71	No	Yes	No	
* Q16. Concerns about the Future*	2.95	1.67	−0.32	6.22	Yes	Yes	Yes	
* Q17. Death and dying*	3.08	1.79	−0.43	6.59	Yes	Yes	Yes	
**Global score of Quality of Life**	**84.18**	**12.60**	**59.48**	**108.88**	**No**	**Yes**	**No**	**0.884**

### Floor and ceiling effects of the WHO-QOL BREF items and domains

Floor and ceiling effects were also examined to assess whether respondents consistently rated items at the lowest or highest ends of the scale (see Table 3), indicating potential limitations in the instrument’s sensitivity. The analysis assumed a floor or ceiling effect if the upper and lower bound of the 95% of confidence interval of responses were in extreme categories. The presence of floor or ceiling effects was observed in some domains, particularly in items related to Pain and discomfort, Energy and fatigue, and certain items within the Spirituality domain. However, none of the domains or the overall global score of quality of life demonstrated a floor or ceiling effect indicating good sensitivity of the tool in the population.

### Reliability analysis of the WHO-QOL BREF domains

Reliability analysis was conducted using Cronbach’s alpha coefficient to assess the internal consistency of the WHO-QOL BREF Table 3. The overall reliability coefficient for the instrument, described as the Global score of Quality of Life was found to be 0.884, indicating high internal consistency. Subscale reliability coefficients ranged from 0.304 to 0.775, with the lowest reliability observed in the Spirituality domain and the highest in the Environment domain. Despite some variations in reliability across domains, the coefficients for the Global score of QOL was 0.884, exceeding the generally accepted threshold of 0.7.

### Correlation and distribution of WHOQOL-HIV BREF domains

**Fig 1:**
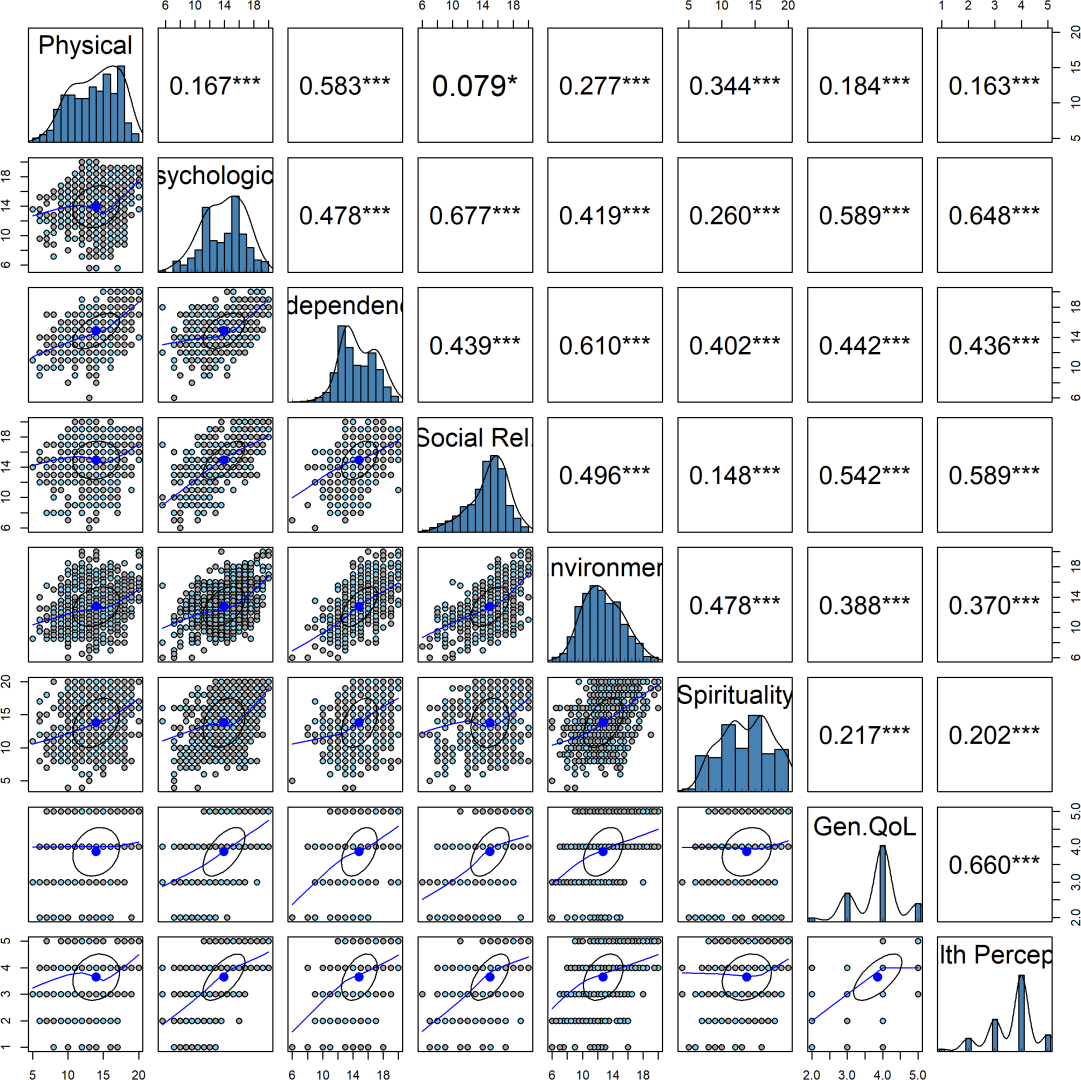
Correlation and distribution of `WHOQOL-HIV BREF domain scores of participants: The pairs-panel diagram shows the distribution of scores for each domain using histograms, correlation matrix, and scatterplot of the relationships. The histogram represents the score range for that domain: The transformed score for the 6 domains ranges from 4–20, while Gen.QOL and Health Perception ranges from 1-5. For the correlation matrix, Pearson’s correlation coefficients (r) are typically categorized as follows: weak (r < 0.3), moderate (r ≥ 0.3 and < 0.7), or strong (r ≥ 0.7). Statistical significance of the correlation is indicated with asterisks: no asterisk (p ≥ 0.05), * = p < 0.01, ** = p < 0.001, *** = p < 0.0001, **** = p < 0.00001. The scatterplot shows the distribution of the association with the central tendency.

The WHOQOL-HIV BREF’s concurrent validity was examined using Pearson’s correlations between domains and general quality of life and health perception **Fig 1**. Results showed that Physical and Psychological domain had a weak to moderate correlation (r = 0.478). Similarly, the Physical and Social Relationships domains showed a weak to moderate positive correlation of 0.496. The correlation between the Physical and Environment domains was also weak to moderate and was r = 0.478. In contrast, the correlation between the Physical and Spirituality domains was weak, at 0.217. However, a moderate positive correlation of 0.660 was observed between the Physical domain and General Quality of Life. **Fig 1** also shows the distribution of the domains of WHOQOL-HIV BREF within the sample, utilizing histograms and scatter plots to represent the range of scores for each domain.

### Confirmatory factor analysis of the WHOQOL-HIV BREF

**Fig 2:**
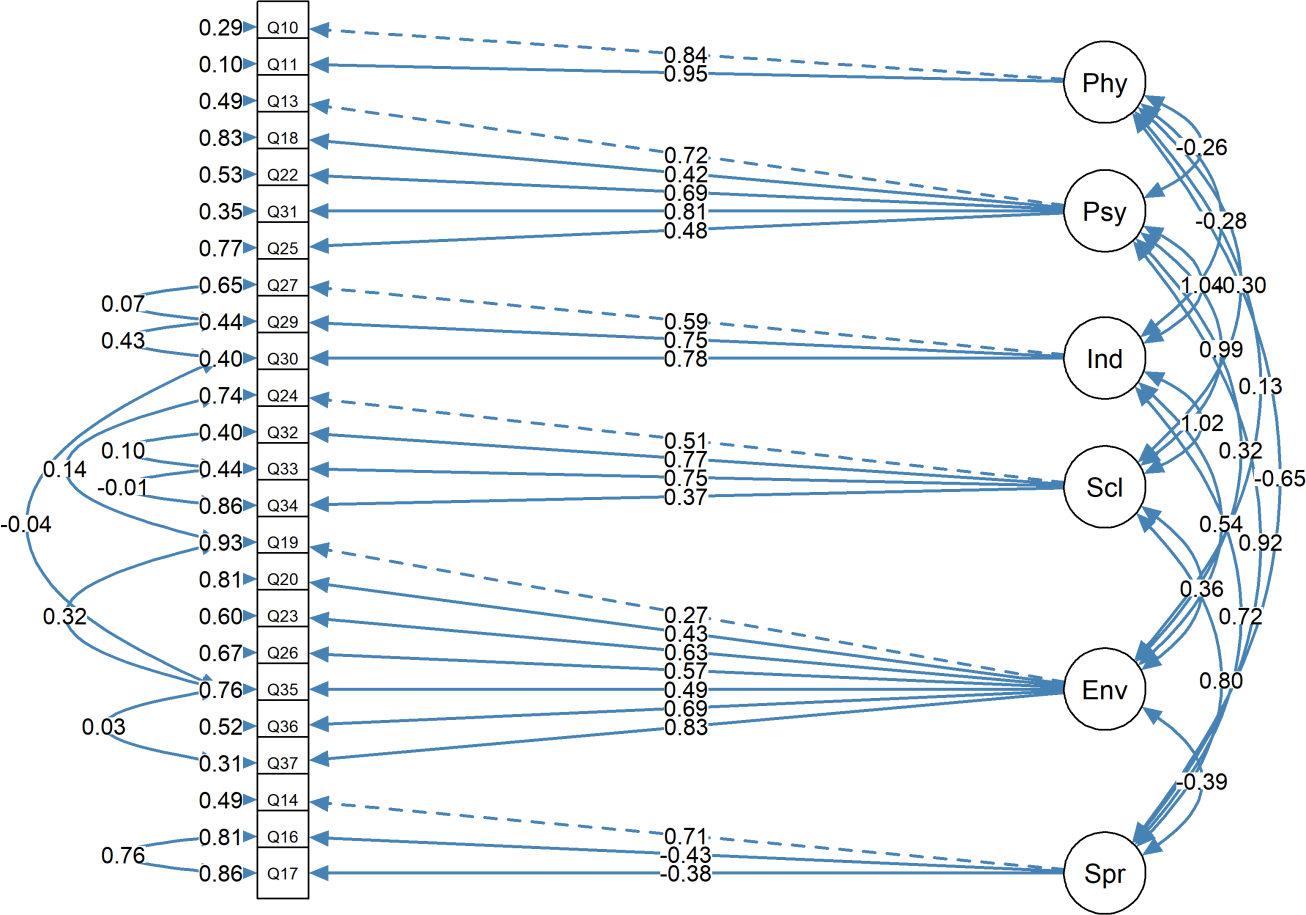
Path diagram of the structure of the WHOQOL-HIV BREF Instrument for the sample based on CFA: Comparative Fit Index (CFI) = 0.807, Tucker-Lewis Index (TLI) = 0.767, RMSEA (TLI) = 0.110, and Standardized Root Mean Square Residual (SRMR) = 0.136. These values are below the generally accepted thresholds for good fit (CFI > 0.95, TLI > 0.95, RMSEA < 0.06 SRMR < 0.08). The factor loadings between the latent variables (domains) and the observed variables are standardized coefficients.

In the Structural Equation Model based on confirmatory factor analysis the result presented in path diagram (**Fig 2**) illustrates the relationship between the latent domain variables and their manifest (observed) variables. The SEM analysis revealed varied contributions of indicators across well-being domains. In the Physical domain, Pain and discomfort and Energy and fatigue showed strong positive factor loadings (β = 0.95 and β = 0.84), while Sleep and rest and Symptoms of PLHIV did not contribute to the model for the sample. The Psychological domain demonstrated medium to high positive loadings for all indicators, including Self-esteem (β = 0.81). Independence was influenced by Mobility, Work capacity, and Activities of daily living (β = 0.59–0.78), while the Social domain was positively associated with Social inclusion, Personal relationships, and Sexual activity (β = 0.37–0.77). Environmental factors such as Financial resources, Recreation, Health and social care, and Transport strongly impacted scores (β = 0.57–0.83). However, in the Spiritual domain, Concerns about the Future and Death and dying had weak negative effects (β = −0.43). The SEM also revealed interconnections among domains, underscoring their collective contribution to overall well-being. However, the fit indices for the structural equation model obtained showed a suboptimal model with the following indices: Comparative Fit Index (CFI) = 0.807, Tucker-Lewis Index (TLI) = 0.767, Root Mean Square Error of Approximation (RMSEA) = 0.110, and Standardized Root Mean Square Residual (SRMR) = 0.136. Given these results, it is important to note a limitation of this SEM model as the model fit fell below commonly accepted thresholds for good fit.

## Discussion

The results of the study assessed the psychometric properties of the QOL tool measured by the WHOQOL-HIV BREF. The reliability results showed generally unsatisfactory reliability of the tool across domains ranging from 0.304 for physical domain to 0.775 for the Environment domain. This finding contrast studies in Tanzania [29], Spain [15], and Thailand [30], which reported acceptable reliability, though these studies used translated and culturally adapted versions rather than the original English tool. Some authors in Ethiopia have reported cross-cultural problems with several items when applying the original WHOQOL-HIV-BREF in Ethiopia. In their work the authors deleted seven items and added context-relevant items because of acceptability/semantic problems and poor psychometric behaviour; one domain (spirituality) showed low internal consistency in the pilot. Generally, the original item set required modification to work reliably in the Ethiopian setting. Additionally, some studies have also highlighted potential limitations in the sensitivity of the WHOQOL-HIV BREF, particularly regarding floor and ceiling effects in certain items, such as those assessing pain and discomfort or financial resources. These effects can cause scores to cluster at the extremes, which may distort domain results and reduce the instrument’s ability to detect changes over time [31]. Similarly, in our study, certain domains also showed limitations, showing clustering of scores at the extremes.

The Structural Equation Model (SEM) results in our study presented the correlation between the latent domains and their respective observed variables, with strong loadings for indicators such as Pain and Energy in the Physical domain and Self-esteem in the Psychological domain. However, certain hypothesized indicators, such as Sleep and PLHIV symptoms in the Physical domain, were not present, suggesting a gap between expected and actual data relationships. This buttresses the idea that cultural or contextual differences is responsible for how respondents prioritize or perceive certain health aspects. Previous studies using exploratory factor analysis have shown mismatch in hypothesized domain factors in the WHOQOL-HIV BREF [32], suggesting peculiarities in the dynamics of the population and location of study. In our study, the high positive factor loadings for the indicators in the independence domain indicate the potential for this domain to be reproducible in the population using the tool, which originally would indicate that PLHIV have reasonably independent lives while on ART [33]. Our SEM model revealed positive factor loadings across domains like Independence, Social well-being, and Environment, but negative loadings were seen in the Spiritual domain. The model’s poor fit indices, despite refinements in the analysis, further indicate limitations in the WHOQO-HIV BREF accurately capturing the phenomenon in the data, reinforcing the peculiarity of study context.

Several studies have shown that translating and adapting the WHOQOL-HIV BREF into local languages and cultural context enhances its performance [32,34,35]. For instance, a study in Pakistan demonstrated that translating the tool into Urdu and adapting it to the local culture improved its relevance and measurement accuracy [32]. In the study, the exploratory factor analysis revealed a five-domain structure, rather than replicating the original six domains, with some domains merging (e.g., level of independence and physical health) in the Pakistani context [32]. In contrast, a study in Spain confirmed the validity and reliability of the Spanish version while mainly preserving the original six-domain structure [34]. These variations highlight the flexibility of the instrument to accommodate cultural differences while maintaining its usefulness in assessing quality of life among people living with HIV.

Our study has some limitations. The WHOQOL-HIV BREF was applied without prior cultural adaptation or validation for the target population, meaning the tool was used in its existing form rather than through a standardized translation and adaptation process. WHO has recommends the following steps for the implementation of translation and cultural adaptation of the WHOQOL-HIV BREF: forward translation, expert panel review, back translation, pretest and cognitive interviews and formulation of the final version [36]. In addition, while exploratory factor analysis is sometimes undertaken when CFA shows poor model fit, we did not proceed in this case. This is because the reliability of the WHOQOL-HIV BREF domains in our data were very low, indicating weak internal consistency. This lack of reliability would make any exploratory factors unstable and difficult to interpret in a meaningful way.

## Conclusion

This study revealed limited structural validity and varied internal consistency in this sample, with some domains of the WHOQOL-HIV BREF performing poorly. These findings reinforce concerns raised by researchers about the use of the tool without proper contextual adaptation. Further research is needed to better understand QOL in the Nigeria population, by refining and validating the QOL assessment tools for the country, to ensure adequate monitoring of the health of PLHIV.

## Supporting information


S1 Data
Survey Data for the WHOQOL-HIV BREF of PLHIV in Bauchi, Nigeria. (CSV)
